# Triglyceride glucose index combined with plaque characteristics as a novel biomarker for cardiovascular outcomes after percutaneous coronary intervention in ST-elevated myocardial infarction patients: an intravascular optical coherence tomography study

**DOI:** 10.1186/s12933-021-01321-7

**Published:** 2021-06-28

**Authors:** Xiaoxiao Zhao, Ying Wang, Runzhen Chen, Jiannan Li, Jinying Zhou, Chen Liu, Peng Zhou, Zhaoxue Sheng, Yi Chen, Li Song, Hanjun Zhao, Hongbing Yan

**Affiliations:** 1 Department of Cardiology, Peking Union Medical College & Chinese Academy of Medical Sciences, Fuwai Hospital, National Center for Cardiovascular Diseases, 167 Beilishi Road, Xicheng District, Beijing, 100037 China; 2 Department of Cardiology, Fuwai Hospital Chinese Academy of Medical Sciences, Shenzhen, China

## Abstract

**Background and aim:**

This prospective study explored plaque morphology according to the underlying culprit lesion pathology (rupture versus erosion) in relation to the triglyceride glucose (TyG) index in patients with acute ST-elevated myocardial infarction (STEMI) who underwent primary percutaneous coronary intervention and optical coherence tomography (OCT) for culprit lesions to elucidate the effects of the TyG index and type of plaque on the incidence of major adverse cardiovascular events (MACEs).

**Methods and outcomes:**

A total of 274 patients with STEMI aged ≥ 18 years who underwent pre-intervention OCT imaging of culprit lesions between March 2017 and March 2019 were enrolled. The TyG index was calculated using the formula ln[fasting TG (mg/dL) × fasting glucose (mg/dL)/2]. Patients with plaque rupture (PR) and plaque erosion (PE) were divided into three groups across the TyG tertiles. MACEs were defined as a composite of all-cause death, myocardial infarction (MI) recurrence, and ischaemic stroke.

In fully adjusted analyses, the middle tertile of TyG was significantly associated with greater rates of MACEs in patients with PR but not in those with PE (relative to the low tertile, HR [hazard ratio], 6.01; 95% confidence interval [CI], 1.25–28.88; P = 0.025). Cox regression models indicated a significantly higher HR for MACEs in patients in the middle tertile of TyG than in those in the low tertile of TyG after full additional adjustment (HR, 5.45; 95% CI, 1.10–27.09; P = 0.038). However, being in the high tertile of TyG independently and significantly increased the risk of major bleeding events among patients with PE (HR, 2.50; 95% CI, 1.11–5.65; P = 0.028). The area under the receiver operating characteristic curve for predicting MACEs to evaluate the diagnostic value of the TyG index combined with the morphological characteristics of plaque after full adjustment was 0.881 (sensitivity = 94.74%, specificity = 78.04%, cut-off level = 0.73). Kaplan–Meier curves were generated for the cumulative incidence of MACEs for up to a median of 1.98 years stratified by tertiles of TyG among the PR and PE subgroups. Among patients with PR, there were significant differences among the tertiles of TyG (p = 0.030).

**Conclusion and relevance:**

Microstructural OCT features of culprit lesions in combination with the TyG index, a surrogate estimate of insulin resistance, can be used in clinical practice to support risk stratification and predict adverse events in patients with STEMI.

**Supplementary Information:**

The online version contains supplementary material available at 10.1186/s12933-021-01321-7.

## Introduction

Acute coronary syndrome (ACS) has been considered the primary cause of mortality and disability in the contemporary era even though superior optimized drug strategies and therapy, including revascularization, have been developed and widely applied [1–3]. The previous literature has demonstrated that type 2 diabetes mellitus (T2DM) is one of the most significant risk factors for cardiovascular disease (CVD) [4]. The crucial mechanism of the pathogenesis of T2DM, insulin resistance (IR), has been widely proven to be strongly correlated with the process of carotid and coronary atherosclerosis and thus results in a worse prognosis during follow-up [5–8]. The triglyceride glucose (TyG) index, a product of triglyceride (TG) and fasting plasma glucose (FPG) levels, has been shown to be a surrogate estimate of IR and a predictor of CVD in both non-diabetic and diabetic patients [9–12]. Optical coherence tomography (OCT), a cross-sectional and high-resolution intravascular imaging technique, allows the acquisition of detailed in vivo images of coronary plaque morphology characteristics, including plaque rupture (PR) and plaque erosion (PE) [13, 14]. However, the prognostic value of the TyG index combined with the morphological characteristics of vulnerable culprit coronary plaques in predicting cardiovascular outcomes has not been fully investigated. Thus, the development of new therapeutic targets and risk reduction strategies to match the risk level of individuals is urgent. The present study aimed to explore these characteristics, particularly PR and PE, in relation to the TyG index in patients with acute ST-elevation myocardial infarction (STEMI) undergoing primary percutaneous coronary intervention (PCI) and elucidate the effects of different TyG index groups based on the type of plaque on the incidence of major adverse cardiovascular events (MACEs) in a prospective cohort of patients who underwent OCT for culprit lesions.

## Methods

### Study population

For this study, a post hoc analysis of the OCTAMI (**O**ptical **C**oherence **T**omography Examination in **A**cute **M**yocardial **I**nfarction) registry was performed, in which 434 consecutive patients hospitalized for STEMI and screened by OCT underwent primary PCI from March 2017 to March 2019 at one of the top-ranked and largest PCI centres in China. The major exclusion criteria were cardiac shock, serious liver dysfunction, allergy to contrast media, severe hepatic and renal insufficiency (estimated glomerular filtration rate [eGFR] < 30 ml/min), congestive heart failure (left ventricular ejection fraction < 50%), contraindication to aspirin or ticagrelor and lesions with characteristics that raised the difficulty and risk of performing OCT (e.g., chronic total occlusion, heavily calcified vessels and left main coronary artery diseases). The definition of STEMI followed the established criteria [15].

The study protocol was conducted in accordance with the principles outlined in the Declaration of Helsinki and was approved by the Ethics Committee of Fuwai Hospital, Peking Union Medical College & Chinese Academy of Medical Sciences, with a waiver of informed consent. Personal information related to the identities of the patients was concealed. Patients who were examined using OCT provided written informed consent specific to the OCT study (Fuwai Hospital OCTAMI Registry, clinical trials.gov: NCT03593928).

### Definition

Hypertension was defined as blood pressure (BP) ≥ 140/90 mmHg at rest over three measurements or a previous diagnosis of hypertension and current use of antihypertensive drugs [16]. Patients were diagnosed with DM if they met one of the following criteria: (i) fasting plasma glucose level ≥ 7.0 mmol/L, (ii) 2-h plasma glucose value ≥ 11.1 mmol/L in the 75-g oral glucose tolerance test (OGTT), and (iii) casual plasma glucose level ≥ 11.1 mmol/L [17]. Dyslipidaemia was defined by any of the following parameters [18]: total cholesterol level ≥ 5.0 mmol/L, low-density lipoprotein cholesterol (LDL-C) level ≥ 3.0 mmol/L, triglyceride level ≥ 1.7 mmol/L, high-density lipoprotein cholesterol (HDL-C) level < 1.2 mmol/L (in women) or < 1.0 mmol/L (in men). Patients who did not meet the standards for never smokers (never smoked in their lifetime) or former light smokers (stopped smoking at least 15 years ago, with ≤ 10 total pack-years of smoking) were considered current smokers [19]. Chronic kidney disease (CKD) was defined as abnormal kidney structure or function for more than 3 months, and end-stage renal disease (ESRD) was the final common pathway for CKD [20]. Body mass index was calculated by dividing weight (kg) by the square of height (m^2^).

Prior PCI: Eligible patients underwent PCI once (including percutaneous transluminal coronary angioplasty and stent implantation).

### OCT image acquisition

Intravascular OCT imaging was performed in accordance with previously described methods [8]. In brief, following the restoration of antegrade coronary blood flow and reduction in the thrombus burden by pre-dilatation and/or thrombus aspiration, OCT images of culprit lesions were obtained by a frequency-domain OCT system (ILUMIEN OPTIS™; St. Jude Medical/Abbott, St. Paul, MN, USA) and a catheter (Dragonfly™; LightLab Imaging, Inc., Westford, MA, USA). To remove the blood from the field of view and achieve a virtually blood-free environment, continuous flushing with contrast media via manual injection directly through the guiding catheter was conducted during the acquisition of coronary blood vessels during imaging. The images of the entire length of culprit vessels were acquired by using a pullback device that moved at 36 mm/s automatically, and cross-sectional images were generated at a rate of 180 frames/s rotationally. The length of the OCT pullback was 75 mm in total and digitally archived.

### Quantitative OCT image analysis

**Fig. 1:**
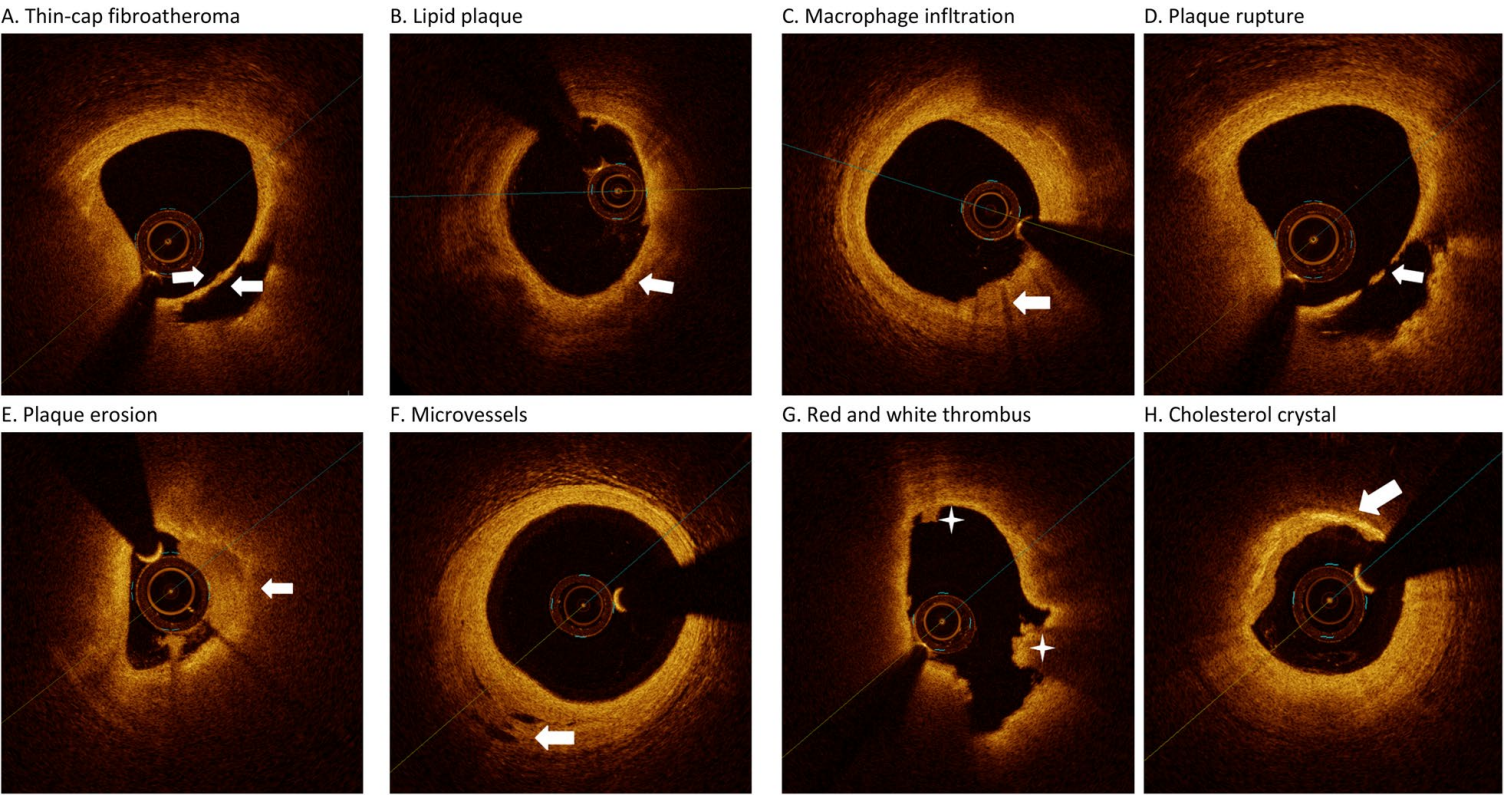
Representative cross-sectional optical coherence tomography images. **A** Thin-cap fibroatheroma was defined as a lipid-rich plaque (lipid identified as signal poor and attenuating) of more than two quadrants of vessel lumen with a fibrous cap (identified as signal rich, or brightly reflecting, with low attenuation) thickness measuring 65 mm or less. (arrow). **B** Lipid plaque (arrow) most often appears as diffusely bordered, signal-poor regions with overlying signal-rich bands. **C** Macrophage infltration (arrow) defned as a signal-rich, distinct or confuent punctate region of higher intensity than background speckle noise that generates remarkable backward shadowing. **D** Plaque rupture identified by disruption of the fibrous cap and cavity formation (asterisk). **E** Plaque erosion identified by the presence of attached thrombus (asterisk) overlying an intact plaque. **F** Microvessels defined as tubule luminal structures that do not generate a signal, with no connection to the vessel lumen (arrow). **G** Red thrombus consists mainly of red blood cells; relevant OCT images are characterized as high-backscattering protrusions with signal free shadowing (asterisk). White thrombi mainly consisted of white blood cells (WBCs) and platelets and were characterized as signal-rich, low-backscattering, billowing projections protruding into the lumen (asterisk). **H** Cholesterol crystal (arrow) identified by linear, highly backscattering structures without remarkable backward shadowing

OCT image analysis was performed using an OCT offline review workstation (Ilumien Optis, St Jude Medical) in a core laboratory by three independent observer investigators (RZ.C., ZX. S. and JN.L.) who were blinded to the angiographic data and clinical presentations of the enrolled patients. They were responsible for screening suitability for culprit plaque evaluation, analysing the characteristics of the plaques and measuring microstructural indices, including maximal lipid arc, minimal fibrous cap thickness and minimal lumen area. Disagreements and inconsistencies among the investigators were resolved by consensus. The entire segment containing the culprit plaque was identified by OCT analysis conducted with the entire OCT pullback. A culprit plaque was defined as segments centred on the culprit lesion and bilaterally extended to more than 5 mm of the normal vessel segment [13]. Based on established criteria [20, 22], thin-cap fibroatheroma was defined as a lipid-rich plaque (lipid identified as signal poor and attenuating) of more than two quadrants of the vessel lumen with a fibrous cap (identified as signal rich, or brightly reflecting, with low attenuation) thickness measuring 65 mm or less (arrow) [21] (Fig. 1A). Lipid plaques (arrow) most often appear as diffusely bordered, signal-poor regions with overlying signal-rich bands (Fig. 1B) [22]. According to International Working Group for Intravascular Optical Coherence Tomography (IWG-IVOCT) Consensus standards [21], macrophage infiltration (arrow) is defined as a signal-rich, distinct or confluent punctate region of higher intensity compared with background speckle noise that generates remarkable backward shadowing (Fig. 1C) [22]. PR was identified by disruption of the fibrous cap and cavity formation (asterisk) (Fig. 1D). PE was identified by the presence of an attached thrombus (asterisk) overlying an intact plaque (Fig. 1E). Microvessels were defined as tubule luminal structures that did not generate a signal, with no connection to the vessel lumen (arrow) [22] (Fig. 1F). Red thrombi consisted mainly of red blood cells; the relevant OCT images were characterized as high-backscattering protrusions with signal-free shadowing (asterisk). White thrombi mainly consisted of white blood cells (WBCs) and platelets and were characterized as signal-rich, low-backscattering, billowing projections protruding into the lumen (asterisk) [8] (Fig. 1G). Cholesterol crystals (arrow) were identified as linear, highly backscattering structures without remarkable backward shadowing (Fig. 1H) [21]. Calcification was defined as the presence of well-delineated, low-backscattering, heterogeneous regions. The lipid arc was measured at 1-mm intervals across the entire lesion, and the largest arc was recorded [22]. The length of the culprit lesion was defined as the span of the entire culprit plaque in the longitudinal view, as measured using OCT [22]. The minimal lumen area was assessed along with the length of the target lesion [22].

### Measurements

Baseline data, including patient clinical demographics such as age, sex, smoking status, history of disease (including hypertension, diabetes, hyperlipidaemia and chronic kidney disease) and PCI, laboratory results, primary PCI procedures, and medical treatments, were obtained from hospital records. Serum levels of fasting plasma glucose and lipid profiles, including TG, total cholesterol (TC), lipase activator (LPA) and HDL-C, were determined by standard laboratory techniques at Fuwai Hospital. The TyG index was computed using the following formula: ln[fasting TG (mg/dL) × FPG (mg/dL)/2] [23]. The definition of TYH was TyG plus Ln0.01129.

### Endpoints and follow-up

MACEs were defined as a composite of all-cause mortality, non-fatal MI and ischaemic cerebrovascular events. Stroke was diagnosed by the presence of rapidly developing focal or widespread brain dysfunction that lasted more than 24 h or caused death, excluding non-vascular causes. Non-fatal MI was diagnosed as the symptom of typical chest pain or changes in typical serial electrocardiograms combined with positive cardiac troponins.

A clinical follow-up was performed after 3 years via direct interviews, telephone calls and hospital discharge records or clinical notes in the event of death, and well-trained physicians and nurses performed the clinical follow-up with the patients enrolled in the OCTAMI registry (median time to follow-up: 23.96 months). The follow-up protocol was approved by obtaining permission from the Institutional Review Board of Fuwai Hospital. Well-trained physicians in charge of the follow-up primary endpoints, including angina pectoris, cardiac death, all-cause death, non-fatal MI, revascularization, heart failure, ischaemic stroke, haemorrhagic apoplexy and bleeding events, identified and extracted the primary endpoints from hospital records, laboratory reports, emergency records, medical records, and clinical notes (required to be sent to our centres). More than two professional physicians blinded to the clinical and angiographic data confirmed the clinical endpoints.

### Statistical analysis

The distribution of outcome variables was assessed using the Kolmogorov–Smirnov test. Continuous data are presented as the median (25th and 75th percentiles: P25, P75) in the case of normal or non-normal distribution. Between-group differences were analysed using an independent sample t-test or the Mann–Whitney U test for normally and non-normally distributed data, respectively. Categorical data are presented as numbers (percentages) and were compared using Pearson’s chi-squared (χ^2^) test or Fisher’s exact test, as appropriate. Multivariable Cox proportional hazards regression models with adjustments for confounding factors were used to assess the associations of the TyG index and PR/PE determined by OCT with MACEs. Kaplan–Meier survival curves were constructed to evaluate the incidence rate of MACEs among the groups according to the optimal tertile point of the TyG index, and discrepancy rates of cumulative events were compared using the log-rank test. Adjustments were made for variables including age; sex; ejection fraction; history of diabetes mellitus, hypertension, hyperlipidaemia, myocardial infarction, PCI and coronary artery bypass graft (CABG); Killip classification; high-sensitivity C-reactive protein (hs-CRP); LDL-C; white blood cell count; platelet count; creatinine; glycated haemoglobin; haemoglobin and the discharge medication prescribed to patients (including aspirin, ticagrelor and clopidogrel). The areas under the receiver operating characteristic curve (ROC), sensitivity, specificity, Youden index and 95% confidence interval (CI) were calculated to evaluate the predictive ability of TyG combined with plaque characteristics (PR&PE) for MACEs.

Statistical analyses were performed using SPSS (version 20.0; IBM Corp., Armonk, NY, USA), R Programming Language X64 4.0.4 (R Foundation for Statistical Computing, Vienna, Austria), and MedCalc version 18.2.1 (MedCalc Software, Ostend, Belgium). Statistical significance was set at P < 0.05, and all P values were two-tailed.

## Results

**Fig. 2:**
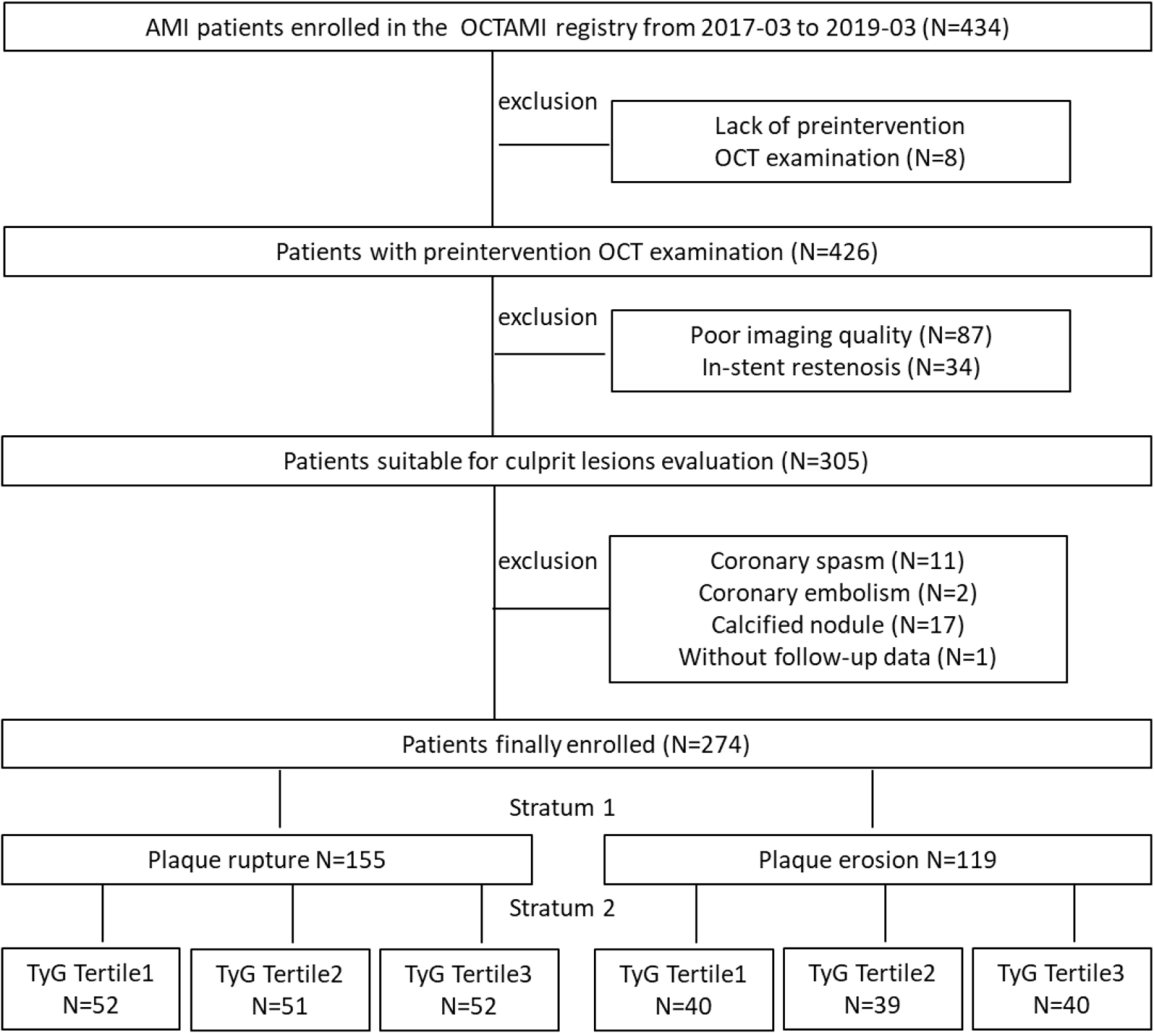
Flow chart 2 Study flow chart. OCTAMI, **O**ptical **C**oherence **T**omography Examination in **A**cute **M**yocardial **I**nfarction; OCT optical coherence tomography, AMI acute myocardial infarction

Among the 434 consecutive patients with STEMI who underwent OCT imaging of native culprit vessels before primary PCI between March 2017 and March 2019, 160 patients who met the major exclusion criteria and one patient lost to follow-up were excluded. Consequently, 274 patients were included in the final analysis. A flow diagram illustrating the study sample selection process is shown in Fig. 2.

### Baseline angiographic and OCT data

Baseline characteristics are shown in Table 1. Among the included patients (80.7% male, mean age 58.0 [range, 50.0–67.0] years), 164 (59.9%), 78 (28.5%), and 236 (86.1%) had hypertension, diabetes mellitus, and hyperlipidaemia, respectively. Some of the participants presented with angina (7.7%), while small subsets of patients died (0.7%) or had recurrent MI (2.9%) and stroke (3.3%). The median values of concordance/discordance were 7.6 mmol/L for FPG and 4.7 for the TYG index. Furthermore, Table 1 summarizes the clinical and angiographic characteristics stratified by PR (n = 119) and PE (n = 155). Patients in the PR group had a higher residual syntax score (5.0 [range, 2.0–9.0]) than those in the PE group [2.0 [range, 0.0–7.0]) (P = 0.006). Furthermore, brain natriuretic peptide levels and platelet counts showed statistically significant, but not clinically relevant, differences. Endpoints and other laboratory examinations were not significantly different among the four groups. Representative OCT images are shown in Fig. 1.

**Table 1:** Baseline clinical characteristics of the study population

Variables	Total (N = 274)	Plaque erosion (N = 119)	Plaque rupture (N = 155)	P value
Age (years)	58.0 (50.0, 67.0)	56.0 (50.0, 65.5)	60.0 (50.0, 67.5)	0.054
Male [%(n)]	221 (80.7)	93 (78.2)	128 (82.6)	0.444
Height (cm)	170.0 (165.0, 173.0)	169.0 (165.0, 173.0)	170.0 (165.0, 173.8)	0.296
Weight (kg)	75.0 (67.0, 82.2)	75.0 (67.0, 80.0)	75.0 (67.2, 84.0)	0.220
Heart rate (beats per minute)	76.0 (65.0, 86.0)	78.0 (66.0, 87.8)	75.0 (65.0, 85.0)	0.357
SBP (mmHg)	121.0 (107.0, 134.0)	120.0 (109.0, 128.8)	122.0 (106.5, 135.5)	0.419
DBP(mmHg)	79.0 ± 12.3	78.9 ± 12.4	79.0 ± 12.4	0.978
Syntax score of base	16.0 (11.0, 22.5)	15.5 (10.0, 22.0)	16.5 (11.0, 22.8)	0.372
Residual syntax score	4.0 (0.0, 8.0)	2.0 (0.0, 7.0)	5.0 (2.0, 9.0)	0.006*
Risk factors				
Hypertension[%(n)]	164 (59.9)	70 (58.8)	94 (60.6)	0.857
Diabetes[%(n)]	78 (28.5)	28 (23.5)	50 (32.3)	0.147
Hyperlipidemia[%(n)]	236 (86.1)	99 (83.2)	137 (88.4)	0.291
Smoking[%(n)]	165 (68.2)	66 (64.1)	99 (71.2)	0.298
Previous PCI[%(n)]	22 ( 8.0)	6 (5)	16 (10.3)	0.171
CKD[%(n)]	4 ( 1.6)	0 (0)	4 (2.8)	0.138
Laboratory examinations				
HDL-cholesterol (mmol/L)	1.1 (0.9, 1.2)	1.1 (1.0, 1.2)	1.1 (0.9, 1.2)	0.177
LDL-cholesterol at (mmol/L)	2.8 ± 0.9	2.8 ± 0.9	2.8 ± 0.8	0.906
Triglycerides (mmol/L)	1.4 (1.0, 2.0)	1.4 (0.9, 2.0)	1.4 (1.0, 2.1)	0.681
LPA (mg/L)	159.1 (71.5, 376.1)	181.0 (65.0, 439.2)	141.0 (75.8, 345.1)	0.282
hs-CRP (mg/L)	6.2 (2.7, 10.9)	6.7 (3.2, 10.9)	5.8 (2.2, 10.9)	0.212
D-dimer (ug/mL)	0.3 (0.2, 0.4)	0.3 (0.2, 0.4)	0.3 (0.2, 0.4)	0.937
TnI (ng/L)	0.9 (0.1, 5.2)	1.5 (0.1, 5.2)	0.4 (0.1, 4.6)	0.041
Peak level of TnI (ng/L)	23.9 (10.8, 46.5)	26.0 (11.3, 49.0)	22.0 (9.7, 41.5)	0.152
BNP (ng/L)	137.3 (47.9, 566.0)	224.5 (75.6, 717.2)	103.0 (39.5, 416.2)	0.024*
Peak level of BNP (ng/L)	1606.0 (633.5, 3122.0)	1463.0 (607.2, 2946.0)	1657.0 (673.8, 3179.0)	0.544
WBC 10^9^/L	9.8 (8.0, 11.9)	10.1 (8.1, 12.7)	9.6 (7.9, 11.4)	0.071
Hemoglobin (g/L)	147.5 (136.0, 156.0)	149.0 (136.0, 155.0)	146.0 (135.0, 156.5)	0.603
Platelet 10^9^/L	222.0 (191.2, 280.8)	241.0 (198.0, 295.5)	212.0 (187.0, 266.0)	0.007*
Crea (umol/L)	79.0 (67.9, 91.8)	75.8 (65.1, 89.5)	82.3 (70.6, 94.1)	0.005
Fasting plasma glucose (mmol/L)	7.6 (6.3, 10.0)	7.5 (6.3, 9.8)	7.7 (6.3, 10.2)	0.351
A1C (%)	6.0 (5.6, 7.1)	6.0 (5.6, 6.8)	6.0 (5.6, 7.4)	0.408
TYG^a^	4.7 ± 0.7	4.6 ± 0.7	4.7 ± 0.8	0.410
TYG index tertiles				
T1, n (%)	–	3.9 ± 0.3	3.9 ± 0.3	-
T2, n (%)	–	4.6 ± 0.2	4.6 ± 0.2	-
T3, n (%)	–	5.5 ± 0.5	5.3 ± 0.4	-
Discharge medication regimen				
Aspirin[%(n)]	265 (96.7)	116 (97.5)	149 (96.1)	0.736
Ticagrelor[%(n)]	139 (50.7)	53 (44.5)	86 (55.5)	0.094
Clopidogrel[%(n)]	135 (49.3)	66 (55.5)	69 (44.5)	0.094
ACEI/ARB[%(n)]	204 (74.5)	86 (72.3)	118 (76.1)	0.558
Beta-Blockers[%(n)]	240 (87.6)	103 (86.6)	137 (88.4)	0.786
Statin[%(n)]	266 (97.1)	116 (97.5)	150 (96.8)	1.000
Proton pump inhibitor[%(n)]	109 (39.8)	52 (43.7)	57 (36.8)	0.300
Oral anticoagulants[%(n)]	6 ( 2.2)	4 (3.4)	2 (1.3)	0.408
Procedural data				
Angiographic findings				
Culprit vessels				0.002*
LAD	131 (47.8)	65 (54.6)	66 (42.6)	
LCX	27 ( 9.9)	17 (14.3)	10 (6.5)	
RCA	116 (42.3)	37 (31.1)	79 (51)	
Coronary artery lesions				0.012*
SVD	66 (24.1)	39 (32.8)	27 (17.4)	
DVD	100 (36.5)	40 (33.6)	60 (38.7)	
TVD	108 (39.4)	40 (33.6)	68 (43.9)	
Pre-TIMI flow				0.755
0	172 (62.8)	79 (66.4)	93 (60)	
1	15 ( 5.5)	6 (5)	9 (5.8)	
2	25 ( 9.1)	10 (8.4)	15 (9.7)	
3	62 (22.6)	24 (20.2)	38 (24.5)	
AHA classification				0.990
A	2 ( 0.7)	1 (0.8)	1 (0.6)	
B1	25 ( 9.2)	11 (9.3)	14 (9)	
B2	38 (13.9)	17 (14.4)	21 (13.5)	
C	208 (76.2)	89 (75.4)	119 (76.8)	
Diameter of lesion (mm)	3.0 (2.8, 3.5)	3.0 (2.8, 3.5)	3.0 (2.8, 3.5)	0.058
Length of lesion (mm)	26.0 (19.0, 37.0)	26.0 (19.0, 34.8)	26.0 (20.0, 40.0)	0.160
Endpoint events				
MACE [%(n)]	19 (6.9)	7 (5.9)	12 (7.7)	0.718
Death [%(n)]	2 (0.70)	0 (0)	2 (1.3)	0.507
Recurrent MI [%(n)]	8 (2.90)	5 (4.2)	3 (1.9)	0.300
Stroke [%(n)]	9 (3.30)	2 (1.7)	7 (4.5)	0.307
Angina [%(n)]	21 (7.70)	8 (6.7)	13 (8.4)	0.776
Heart failure [%(n)]	6 (2.20)	5 (4.2)	1 (0.6)	0.089

Continuous data are presented as mean ± standard deviation (SD) or median (25th, 75th percentiles). Categorical data are presented as number (%)

SBP, systolic blood pressure; DBP, diabetes blood pressure; PCI, percutaneous coronary intervention; CKD, chronic kidney disease; HDL, high density lipoprotein; LDL, low density lipoprotein; LPA, lipse activator; hs-CRP, high sensitive C-reactive protein; BNP, type B natriuretic peptide; WBC, White blood cell; Crea,creatinine; ACEI, angiotensin-converting enzyme inhibitor; ARB, angiotensin receptor blocker; TnI, troponin; LAD, left anterior descending artery; LCX, left circumfex artery; RCA, right coronary artery; DVD, double vessel disease; SVD, single vessel disease; TVD, triple vessel disease. pre-TIMI, previous of procedural thrombolysis in myocardial infarction flow grade; AHA, American Heart Association; MACE, major adverse cardiovascular events; MI, myocardial infarction. ^a^, TYH, defined as TyG plus LN0.01129; TyG, triglyceride glucose; A1C, Glycated hemoglobin; T, tertiles of TYG

*P < 0.05

A comparison of plaque characteristics based on OCT findings divided by tertiles of the TyG index is presented in Table 2. The frequencies of microstructural features, such as plaque morphology, thin-cap fibroatheroma, healing plaques, calcification, microcalcification, microvessels, cholesterol crystals, and thrombus, were similar among the three groups, as were quantitative parameters, such as the minimal lumen area, minimal fibrous cap thickness, and maximal lipid arc.

**Table 2:** Optical coherence tomography characteristics

Variables	Total (N = 274)	TyG Tertiles 1	TyG Tertiles 2	TyG Tertiles 3	P^#^_for overall_
Plaque morphology					0.796
Plaque rupture[%(n)]	119 (43.4)	39 (42.9)	42 (46.2)	38 (41.3)	
Intact fibrous cap[%(n)]	155 (56.6)	52 (57.1)	49 (53.8)	54 (58.7)	
Lipid-rich plaque[%(n)]	262 (95.6)	89 (97.8)	83 (91.2)	90 (97.8)	0.053
Fibrous plaque[%(n)]	78 (28.5)	19 (20.9)	32 (35.2)	27 (29.3)	0.100
Mixed plaque [%(n)]	60 (21.9)	24 (26.4)	20 (22.0)	16 (17.4)	0.340
Healing plaque[%(n)]	55 (20.1)	12 (13.2)	20 (22.0)	23 (25.0)	0.117
Calcification[%(n)]	140 (51.1)	47 (51.6)	41 (45.1)	52 (56.5)	0.298
Micro-calcification[%(n)]	136 (49.6)	45 (49.5)	40 (44)	51 (55.4)	0.299
Macrophage[%(n)]	149 (54.4)	53 (58.2)	47 (51.6)	49 (53.3)	0.648
Microvessels[%(n)]	48 (17.5)	16 (17.6)	16 (17.6)	16 (17.4)	0.999
Cholesterol crystal[%(n)]	22 ( 8.0)	10 (11)	4 (4.4)	8 (8.7)	0.251
Thrombus[%(n)]	271 (98.9)	91 (100)	89 (97.8)	91 (98.9)	0.547
Minimal FCT, um	100.0 (60.0, 120.0)	100.0 (70.0, 120.0)	100.0 (70.0, 140.0)	90.0 (60.0, 120.0)	0.402
Maximal lipid arc, °	360.0 (248.0, 360.0)	360.0 (252.0, 360.0)	317.0 (240.0, 360.0)	360.0 (251.2, 360.0)	0.256
MLA, mm2	1.7 (1.4, 2.2)	1.6 (1.4, 2.1)	1.8 (1.4, 2.4)	1.7 (1.3, 2.2)	0.310

Continuous data are presented as median (interquartile range). Categorical variables are presented as number (%)

TyG, triglyceride glucose; TCFA, thin-cap fibroatheroma; FCT fibrous cap thickness; MLA minimal lumen area

*P < 0.05,

^#^ P for overall means statistical analysis among four groups

### Findings of Cox regression models in subgroups

Table 3 describes the fully adjusted multivariable relationships between MACEs stratified according to TyG levels and culprit plaque characteristics revealed by OCT during treatment. In the group with higher TyG levels, the risk of MACEs decreased significantly during grouping (≥ median vs. < median, median of TyG=9.1424) in the overall population (HR, 0.29; 95% CI, 0.09–0.93; P = 0.037) but not in patients with PR (HR, 0.91; 95% CI, 0.35–2.38; P = 0.847). However, when stratifying the overall population according to plaque characteristics (PR vs. PE) and tertiles of TyG levels, the middle tertile of the TyG index was significantly associated with MACEs only when the characteristic was PR (relative to tertile 1, the HR for MACEs in tertile 2 was 6.01; 95% CI, 1.25–28.88; adjusted P = 0.025) after full adjustment (sex, age, history of hypertension, history of hyperlipidaemia, history of diabetes mellitus, white blood cell count, heart rate, residual syntax score, and C-reactive protein level). However, in the setting of PE, TyG levels were not associated with a greater risk of clinical events.

**Table 3:** Associations between MACE stratified according to type of plaque and triglyceride glucose index tertiles

Population	Kaplan–Meier estimate, No. / total No. (%)	Crude HR (95% CI)	MACE, Crude P value (95% CI)	MACE, Adjusted HR (95% CI)^a^	MACE, Adjusted P value
Overall OCT population	19 ( 6.90)	NA	NA	NA	NA
Plaque erosion	7 (5.90)	1 [Reference]	NA	1 [Reference]	NA
Plaque rupture	12 (7.70)	1.27 (0.50,3.23)	0.614	0.91 (0.35,2.38)	0.847
Overall TyG per SD	19 ( 6.90)	0.52(0.32,0.86)	0.014*	0.46(0.25,0.85)	0.014*
< Median	14 (10.20)	1 [Reference]	NA	1 [Reference]	NA
≥ Median	5 (3.60)	0.33 (0.12,0.93)	0.036*	0.29 (0.09,0.93)	0.037*
Plaque rupture					
TyG per SD	12 ( 7.7)	0.669(0.36,1.26)	0.212	0.83(0.38,1.84)	0.653
T1	3 (5.80)	1 [Reference]	NA	1 [Reference]	NA
T2	8 (15.70)	2.77 (0.74,10.46)	0.132	6.01 (1.25,28.88)	0.025*
T3	1 (1.90)	0.33 (0.03,3.13)	0.331	0.90 (0.08,10.47)	0.936
Trend test	NA	0.77 (0.38,1.55)	0.464	1.12 (0.48,2.62)	0.799
Plaque erosion					
TyG per SD	7 (5.90)	0.35(0.15,0.80)	0.013*	0.14(0.03,0.82)	0.029*
T1	5 (12.50)	1 [Reference]	NA	1 [Reference]	NA
T2	1 (2.60)	0.20 (0.02,1.74)	0.146	0.14 (0.01,2.54)	0.186
T3	1 (2.50)	0.19 (0.02,1.62)	0.128	0.09 (0.00,2.00)	0.126
Trend test	NA	0.37 (0.12,1.13)	0.080	0.27 (0.05,1.45)	0.128

HR, hazard ratio; CI, confidential interval; MACE, major adverse cardiovascular events (cardiovascular death, myocardial infarction, or stroke); NA, not applicable; T, tertiles of TyG; TyG, triglyceride glucose index.

^a^Adjusted variables, gender, age, history of hypertension, history of hyperlipidemia, history of diabetes mellitus, white blood cell, heart rate, residual syntax score and C-reaction protein

**Fig. 3:**
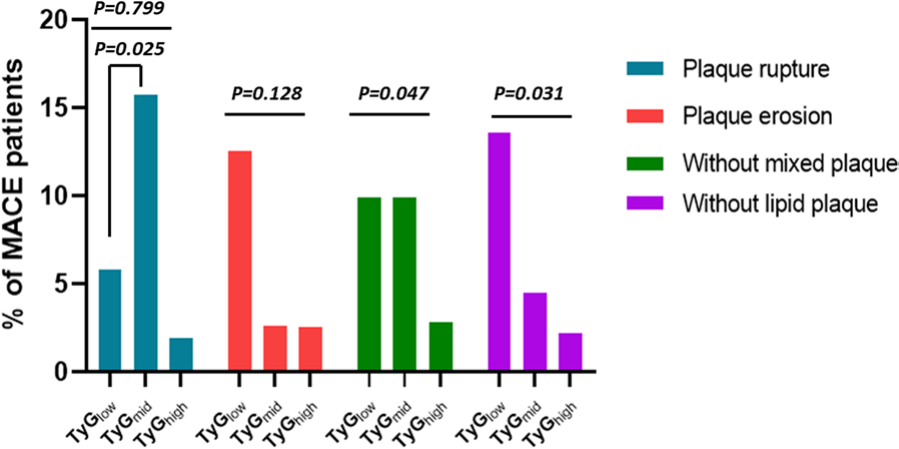
Bar graphs of optical coherence tomography findings of coronary plaques between groups. Comparisons of the incidence of plaque rupture, showed significant differences between patients in TyG_low_ and TyG_mid_. Comparisons of the incidence of patients without mixed plaque and patients without lipid plaque showed significant differences between patients in the in TyG_low_, TyG_mid_ and TyG_high_. However, there are no significant differences among patients with plaque erosion

**Fig. 4:**
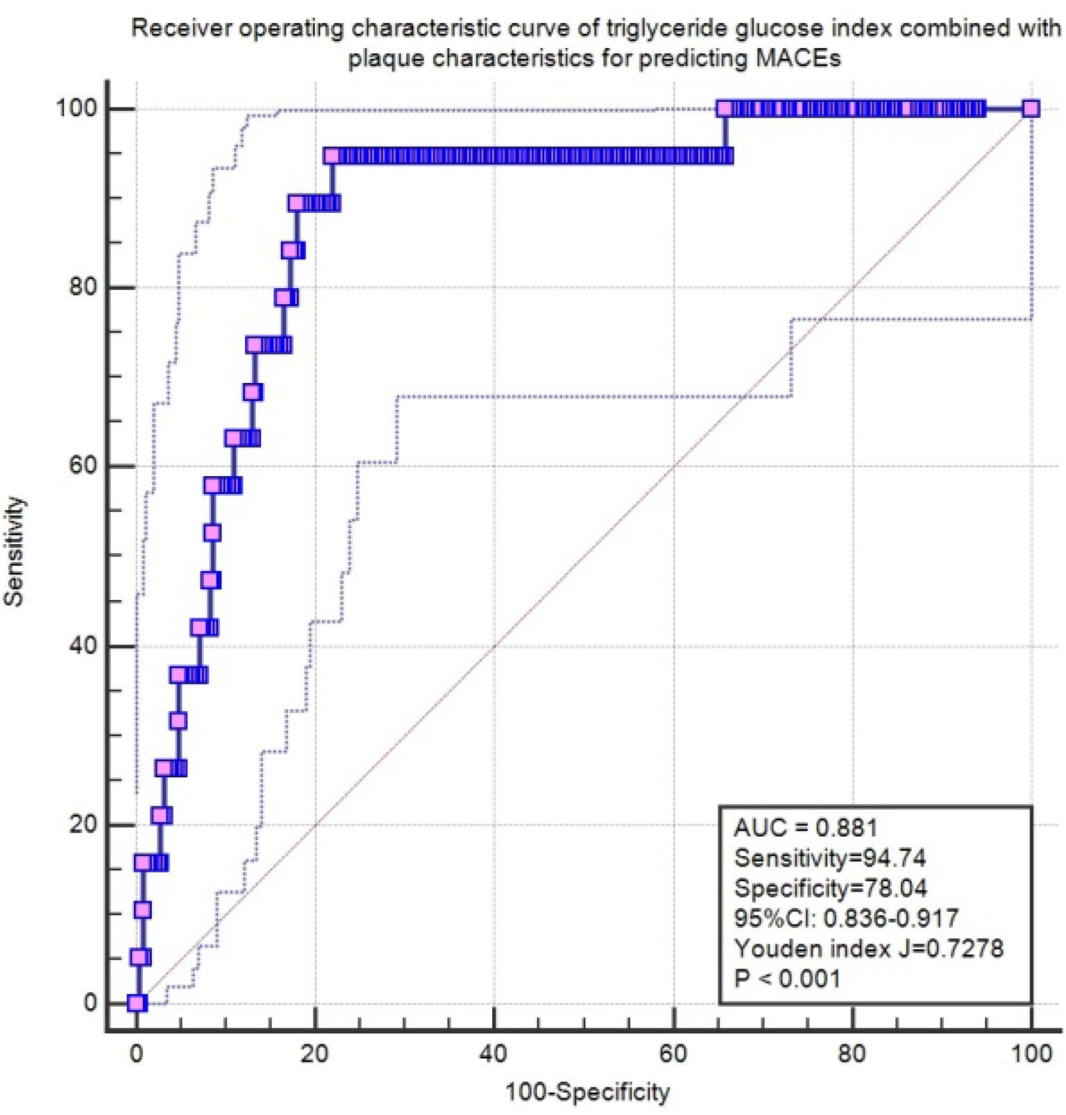
Receiver operating characteristic curve of triglyceride glucose index combined with plaque characteristics for predicting MACES. The area under the ROC was 0.88 (95% CI, 0.84–0.92; this figure). The Youden index was 0.73 and corresponding sensitivity and specificity were 94.74% and 78.04%. MACEs, major adverse cardiovascular events; AUC, areas under the ROC curve; CI, 95% confidence interval

**Fig. 5:**
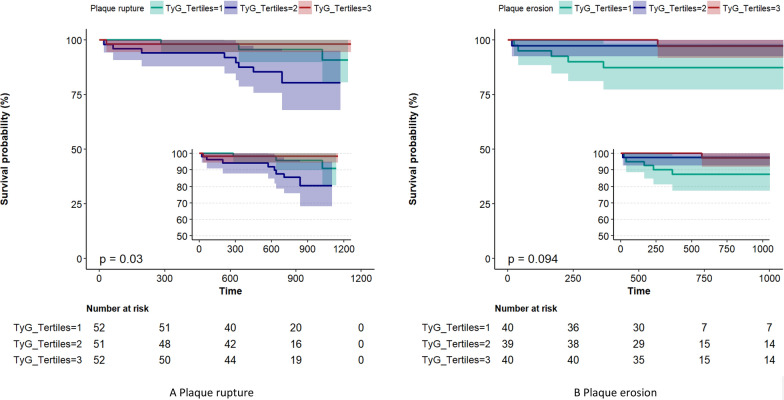
Kaplan-Meier curves showing cumulative MACE rates for up to median 1.98 years stratified by the tertiles level of TyG characteristic among patients with plaque rupture and erosion. A Kaplan–Meier curves showing cumulative MACE rates stratified by the level of TyG among patients with plaque rupture. TyG_Tertiles = 1 represents the patients with low level of TyG among patients with plaque rupture. TyG_Tertiles = 2 represents the patients with mudium level of TyG among patients with plaque rupture. TyG_Tertiles = 3 represent the patients with high level of TyG among patients with plaque rupture. B Kaplan–Meier curves showing cumulative MACE rates stratified by the level of TyG among patients with plaque erosion. TyG_Tertiles = 1 represents the patients with low level of TyG among patients with plaque erosion. TyG_Tertiles = 2 represents the patients with mudium level of TyG among patients with plaque erosion. TyG_Tertiles = 3 represent the patients with high level of TyG among patients with plaque erosion. MACE, major adverse cardiovascular events; TyG, TyG triglyceride glucose

Table 4 shows the crude and adjusted multivariable relationships between MACEs stratified according to tertile levels of the TyG index with PR and PE among the subgroups (Tables 4 and 5, respectively). In patients with PR, the middle tertile of TyG index levels was associated with a higher cumulative incidence of MACEs over time (HR, 5.45; 95% CI, 1.10–27.09; P = 0.038) after full adjustment. Nevertheless, in patients with PE (Table 5), increasing tertiles of TyG index levels were associated with a stepwise lower incidence of MACEs over time (HR, 0.14; 95% CI, 0.02–0.92; P for trend = 0.041). Notably, in the fully adjusted Cox regression models of the PE group, increasing tertiles of TyG index levels were associated with incidence of bleeding risk (HR, 1.59; 95% CI, 1.06–2.39; P for trend = 0.027). Table 6 shows the association of the TyG index with MACEs in enrolled patients according to the subgroup of OCT characteristics. In the subgroup without mixed plaques, the high tertile of the TyG index was a protective factor against the incidence of MACEs compared with the low tertile of the TyG index in the fully adjusted Cox regression models (HR, 0.17; 95% CI, 0.03–0.99; P = 0.048) (Fig. 3). However, no significant differences were found among the other OCT subgroups. ROC analysis was performed to evaluate the diagnostic value of TyG combined with the morphological characteristics of plaques (PR&PE) in predicting MACEs. The area under the ROC curve was 0.88 (95% CI, 0.84–0.92; Fig. 4). The cut-off threshold (Youden index) was 0.73 to generate the maximum sensitivity and specificity in predicting MACEs. The corresponding sensitivity and specificity were 94.74% and 78.04%, respectively. Figure 5 shows the Kaplan–Meier curves for the cumulative incidence of MACEs for up to a median of 1.98 years stratified by tertiles of TyG index levels among the PR and PE subgroups. Among patients with PR, the K-M curve showed significant differences between the tertiles of TyG index levels (p = 0.030). However, the difference was not significant among the patients with PE (p = 0.094).

**Table 4:** Association between separate endpoints survival and groups which divided by TyG in patients with plaque rupture

	Crude model		Adjust model I		Adjust model II		Adjust model III	
Group	crude HR(95%CI)	crude P value	Adj I. HR(95%CI)	Adj. P value	Adj II. HR(95%CI)	Adj. P value	Adj III. HR(95%CI)	Adj. P value
MACE								
1	1 (reference)	1 (reference)	1 (reference)	1 (reference)	1 (reference)	1 (reference)	1 (reference)	1 (reference)
2	2.77 (0.74,10.46)	0.132	3.53 (0.88,14.25)	0.076	3.29 (0.74,14.68)	0.119	5.45 (1.1,27.09)	0.038*
3	0.33 (0.03,3.13)	0.331	0.48 (0.05,4.78)	0.528	0.48 (0.04,5.25)	0.545	0.47 (0.04,4.95)	0.529
Trend test	0.77 (0.38,1.55)	0.464	0.88 (0.41,1.87)	0.737	0.83 (0.36,1.92)	0.664	0.84 (0.38,1.89)	0.678
Revascularization								
1	1 (reference)	1 (reference)	1 (reference)	1 (reference)	1 (reference)	1 (reference)	1 (reference)	1 (reference)
2	0.76 (0.28,2.04)	0.582	0.82 (0.29,2.33)	0.709	0.48 (0.15,1.51)	0.208	0.52 (0.16,1.73)	0.288
3	0.98 (0.39,2.47)	0.967	1.13 (0.42,3.02)	0.806	0.56 (0.15,2.14)	0.398	0.64 (0.16,2.64)	0.540
Trend test	0.99(0.612,1.60)	0.968	1.07 (0.65,1.78)	0.787	0.74 (0.37,1.49)	0.399	0.80 (0.38,1.66)	0.546
Angina								
1	1 (reference)	1 (reference)	1 (reference)	1 (reference)	1 (reference)	1 (reference)	1 (reference)	1 (reference)
2	0.17 (0.02,1.37)	0.095	0.19 (0.02,1.65)	0.131	0.15 (0.02,1.42)	0.099	0.23 (0.02,2.37)	0.217
3	1.01 (0.33,3.13)	0.987	1.23 (0.36,4.22)	0.737	1.02 (0.22,4.77)	0.980	2.92 (0.48,17.62)	0.242
Trend test	1.01(0.52,1.96)	0.988	1.15 (0.57,2.31)	0.691	0.98 (0.41,2.32)	0.961	1.67 (0.64,4.34)	0.294
Bleeding events								
1	1 (reference)	1 (reference)	1 (reference)	1 (reference)	1 (reference)	1 (reference)	1 (reference)	1 (reference)
2	1.09 (0.63,1.86)	0.765	1.1 (0.63,1.93)	0.728	1.12 (0.61,2.06)	0.713	1.15 (0.60,2.20)	0.669
3	0.51 (0.27,0.97)	0.039*	0.47 (0.25,0.92)	0.027*	0.45 (0.20,0.98)	0.045*	0.45 (0.20,1.04)	0.062
Trend test	0.74 (0.56,0.99)	0.045*	0.72 (0.53,0.97)	0.031*	0.71 (0.49,1.01)	0.060	0.70 (0.48,1.04)	0.075

Data presented are HRs and 95% CI. Adjust I model adjusts for sex and age; Adjust II model adjusts for adjust I plus ejection fraction, smoke, hypertension, hyperlipidemia, diabetes mellitus; Adjust III model adjusts for adjust II + creatine kinase, heart rate and C-reactive protain

Adj., adjusted; MACE, major adverse cardiovascular events; HR, hazard ratio; CI, confidence interval

*P < 0.05

**Table 5:** Association between separate endpoints survival and groups which divided by TyG in patients with plaque erosion

	Crude model		Adjust model I		Adjust model II		Adjust model III	
Group	crude HR(95%CI)	crude P value	Adj I. HR(95%CI)	Adj. P value	Adj II. HR(95%CI)	Adj. P value	Adj III. HR(95%CI)	Adj. P value
MACE								
1	1 (reference)	1 (reference)	1 (reference)	1 (reference)	1 (reference)	1 (reference)	1 (reference)	1 (reference)
2	0.20 (0.02,1.74)	0.146	0.12 (0.01,1.70)	0.118	0.69 (0.0,741.76)	0.916	0.09 (0.00,4.23)	0.218
3	0.19 (0.02,1.62)	0.128	0.13 (0.01,1.18)	0.070	0.07 (0.00,1.79)	0.106	0.02 (0.00,0.91)	0.045*
Trend test	0.37 (0.12,1.13)	0.080	0.30 (0.09,1.01)	0.051	0.26 (0.05,1.28)	0.097	0.14 (0.02,0.92)	0.041*
Revascularization								
1	1 (reference)	1 (reference)	1 (reference)	1 (reference)	1 (reference)	1 (reference)	1 (reference)	1 (reference)
2	0.76 (0.17,3.40)	0.718	0.75 (0.17,3.37)	0.705	0.77 (0.13,4.67)	0.777	0.73 (0.11,4.71)	0.737
3	1.21 (0.32,4.51)	0.778	1.24 (0.32,4.71)	0.756	0.98 (0.23,4.11)	0.979	0.93 (0.21,4.12)	0.922
Trend test	1.11 (0.56,2.23)	0.761	1.13 (0.56,2.28)	0.742	0.99 (0.48,2.05)	0.975	0.97 (0.45,2.07)	0.933
Angina								
1	1 (reference)	1 (reference)	1 (reference)	1 (reference)	1 (reference)	1 (reference)	1 (reference)	1 (reference)
2	0.51 (0.09,2.76)	0.431	0.41 (0.08,2.27)	0.309	0.37 (0.04,3.08)	0.357	0.29 (0.03,3.06)	0.304
3	0.49 (0.09,2.67)	0.408	0.38 (0.07,2.13)	0.274	0.19 (0.02,1.73)	0.140	0.19 (0.01,2.42)	0.201
Trend test	0.68 (0.28,1.62)	0.380	0.59 (0.24,1.45)	0.250	0.42 (0.14,1.27)	0.125	0.41 (0.12,1.43)	0.163
Bleeding events								
1	1 (reference)	1 (reference)	1 (reference)	1 (reference)	1 (reference)	1 (reference)	1 (reference)	1 (reference)
2	1.45 (0.72,2.92)	0.295	1.43 (0.71,2.89)	0.313	1.42 (0.63,3.23)	0.397	1.46 (0.63,3.39)	0.379
3	1.80 (0.93,3.50)	0.083	1.75 (0.89,3.41)	0.102	2.16 (1.00,4.67)	0.049*	2.50 (1.11,5.65)	0.028*
Trend test	1.33 (0.96,1.85)	0.083	1.31 (0.95,1.82)	0.103	1.47 (1.00,2.16)	0.047*	1.59 (1.06,2.39)	0.027*

Data presented are HRs and 95% CI. Adjust I model adjusts for sex and age; Adjust II model adjusts for adjust I plus ejection fraction, smoke, hypertension, hyperlipidemia, diabetes mellitus; Adjust III model adjusts for adjust II + creatine kinase, heart rate and C-reactive protain

Adj., adjusted; MACE, major adverse cardiovascular events; HR, hazard ratio; CI, confidence interval

*P < 0.05

**Table 6:** Association of TyG with MACE in enrolled patients according to subgroup of characteristics by OCT

Variables	MACE /Total	Crude model		Adjusted model I		Adjust model II		Adjust model III	
		crude HR(95%CI)	crude P value	Adj I. HR(95%CI)	Adj. P value	adj. HR(95%CI)	Adj. P value	adj. HR(95%CI)	Adj. P value
Without Macrophage									
TyG per SD	6 ( 4.8)	0.30(0.10,0.88)	0.029*	0.38(0.12,1.24)	0.083	0.34(0.11,1.07)	0.065	0.36(0.09,1.38)	0.136
TyG_low_	3 (7.1)	1(reference)	1(reference)	1(reference)	1(reference)	1(reference)	1(reference)	1(reference)	1(reference)
TyG_mid_	3 (7.3)	1.03 (0.21,5.12)	0.969	1.43 (0.26,8.01)	0.684	1.36 (0.22,8.32)	0.736	4.75 (0.32,70.49)	0.258
TyG_high_	0 (0)	0.00 (0.00,Inf)	0.999	0.00 (0.00,Inf)	0.999	0.00 (0.00,Inf)	0.998	0.00 (0.00,Inf)	0.999
Trend test	NA	0.43 (0.14,1.35)	0.149	0.54 (0.16,1.83)	0.325	0.45 (0.1,1.97)	0.292	0.60 (0.13,2.79)	0.517
With Macrophage									
TyG per SD	13 ( 8.7)	0.63(0.35,1.15)	0.131	0.64(0.32,1.25)	0.190	0.52(0.24,1.15)	0.106	0.51(0.23,1.16)	0.111
TyG_low_	5 (10)	1(reference)	1(reference)	1(reference)	1(reference)	1(reference)	1(reference)	1(reference)	1(reference)
TyG_mid_	6 (12.2)	1.2 (0.37,3.92)	0.767	1.25 (0.34,4.6)	0.737	0.99(0.23,4.22)	0.990	1.03 (0.23,4.54)	0.972
TyG_high_	2 (4)	0.36 (0.07,1.86)	0.222	0.38 (0.07,2.18)	0.278	0.32 (0.05,2.12)	0.239	0.31 (0.05,2.12)	0.233
Trend test	NA	0.67 (0.34,1.32)	0.250	0.66 (0.31,1.42)	0.288	0.59 (0.25,1.38)	0.220	0.57 (0.24,1.36)	0.207
Without Calcification									
TyG per SD	10 ( 7.5)	0.52(0.27,1.02)	0.058	0.48(0.23,1.02)	0.056	0.37(0.16,0.86)	0.021*	0.37(0.15,0.93)	0.035*
TyG_low_	5 (11.1)	1(reference)	1(reference)	1(reference)	1(reference)	1(reference)	1(reference)	1(reference)	1(reference)
TyG_mid_	4 (9.1)	0.88 (0.24,3.28)	0.848	0.91 (0.23,3.52)	0.889	1.18 (0.27,5.15)	0.829	1.42 (0.3,6.74)	0.659
TyG_high_	1 (2.2)	0.19 (0.02,1.66)	0.134	0.20 (0.02,1.77)	0.147	0.08 (0.01,1.02)	0.052	0.11 (0.01,1.41)	0.089
Trend test	NA	0.53 (0.23,1.21)	0.131	0.53 (0.23,1.26)	0.152	0.41 (0.16,1.05)	0.063	0.47 (0.18,1.2)	0.113
With Calcification									
TyG per SD	9 ( 6.4)	0.55(0.26,1.15)	0.113	0.46(0.17,1.25)	0.129	0.42(0.15,1.18)	0.100	0.41(0.14,1.21)	0.106
TyG_low_	4 (8.5)	1(reference)	1(reference)	1(reference)	1(reference)	1(reference)	1(reference)	1(reference)	1(reference)
TyG_mid_	4 (8.7)	0.93 (0.23,3.73)	0.920	0.79 (0.16,3.79)	0.765	0.84 (0.16,4.32)	0.837	0.90 (0.17,4.79)	0.899
TyG_high_	1 (2.1)	0.23 (0.03,2.07)	0.191	0.23 (0.02,2.38)	0.219	0.18 (0.02,1.98)	0.162	0.20 (0.02,2.41)	0.207
Trend test	NA	0.57 (0.24,1.34)	0.196	0.53 (0.2,1.44)	0.217	0.49 (0.18,1.34)	0.167	0.52 (0.18,1.49)	0.223
Without Micro-calcification									
TyG per SD	10 ( 7.2)	0.53(0.27,1.04)	0.065	0.50(0.23,1.07)	0.074	0.39(0.17,0.91)	0.028*	0.40(0.16,0.98)	0.043*
TyG_low_	4 (8.7)	1(reference)	1(reference)	1(reference)	1(reference)	1(reference)	1(reference)	1(reference)	1(reference)
TyG_mid_	5 (10.9)	1.38 (0.37,5.14)	0.631	1.50 (0.39,5.82)	0.557	1.96 (0.43,8.93)	0.384	2.38 (0.47,11.94)	0.293
TyG_high_	1 (2.2)	0.25 (0.03,2.21)	0.211	0.26 (0.03,2.47)	0.244	0.13 (0.01,1.63)	0.114	0.17 (0.01,2.29)	0.180
Trend test	NA	0.63 (0.29,1.39)	0.255	0.65 (0.29,1.49)	0.309	0.52 (0.21,1.28)	0.155	0.57 (0.23,1.45)	0.239
With Micro-calcification									
TyG per SD	9 ( 6.6)	0.54(0.26,1.13)	0.102	0.46(0.17,1.24)	0.124	0.42(0.15,1.20)	0.106	0.41(0.14,1.21)	0.108
TyG_low_	4 (8.9)	1(reference)	1(reference)	1(reference)	1(reference)	1(reference)	1(reference)	1(reference)	1(reference)
TyG_mid_	4 (8.9)	0.91 (0.23,3.62)	0.888	0.77 (0.16,3.67)	0.742	0.80 (0.16,4.11)	0.794	0.85 (0.16,4.5)	0.851
TyG_high_	1 (2.2)	0.22 (0.03,2.01)	0.182	0.23 (0.02,2.39)	0.220	0.18 (0.02,1.99)	0.162	0.20 (0.02,2.33)	0.200
Trend test	NA	0.56 (0.24,1.32)	0.185	0.54 (0.2,1.45)	0.219	0.49 (0.18,1.35)	0.167	0.51 (0.18,1.47)	0.213
Without lipid plaque									
TyG per SD	9 ( 6.8)	0.36(0.17,0.74)	0.006*	0.40(0.18,0.86)	0.020*	0.19(0.06,0.58)	0.004*	0.20(0.07,0.59)	0.003*
TyG_low_	6 (13.6)	1(reference)	1(reference)	1(reference)	1(reference)	1(reference)	1(reference)	1(reference)	1(reference)
TyG_mid_	2 (4.5)	0.32 (0.07,1.6)	0.166	0.3 (0.06,1.58)	0.156	0.31 (0.06,1.70)	0.179	0.22 (0.04,1.29)	0.093
TyG_high_	1 (2.2)	0.15 (0.02,1.24)	0.078	0.2 (0.02,1.77)	0.149	0.15 (0.02,1.41)	0.097	0.09 (0.01,1.05)	0.055
Trend test	NA	0.37 (0.14,0.98)	0.045*	0.4 (0.14,1.14)	0.085	0.37 (0.13,1.04)	0.058	0.27 (0.09,0.89)	0.031*
With lipid plaque									
TyG per SD	10 ( 7.1)	0.75(0.38,1.48)	0.402	0.81(0.38,1.72)	0.578	0.71(0.28,1.79)	0.465	0.74(0.29,1.90)	0.536
TyG_low_	2 (4.3)	1(reference)	1(reference)	1(reference)	1(reference)	1(reference)	1(reference)	1(reference)	1(reference)
TyG_mid_	7 (14.9)	3.54 (0.73,17.04)	0.115	4.12 (0.79,21.54)	0.094	2.79 (0.46,16.95)	0.265	6.58 (0.76,56.95)	0.087
TyG_high_	1 (2.1)	0.49 (0.04,5.42)	0.562	0.67 (0.06,7.84)	0.751	0.47 (0.03,6.57)	0.578	0.65 (0.04,10.39)	0.763
Trend test	NA	0.85 (0.4,1.83)	0.683	0.94 (0.4,2.17)	0.878	0.72 (0.27,1.93)	0.513	0.74 (0.28,1.99)	0.557
Without mixed plaque									
TyG per SD	16 ( 7.5)	0.56(0.33,0.97)	0.040*	0.59(0.32,1.06)	0.078	0.45(0.34,0.88)	0.019*	0.43(0.22,0.84)	0.013*
TyG_low_	7 (9.9)	1(reference)	1(reference)	1(reference)	1(reference)	1(reference)	1(reference)	1(reference)	1(reference)
TyG_mid_	7 (9.9)	0.96 (0.34,2.74)	0.939	0.95 (0.29,3.06)	0.925	0.81 (0.24,2.74)	0.736	0.78 (0.22,2.70)	0.691
TyG_high_	2 (2.8)	0.26 (0.05,1.26)	0.095	0.30 (0.06,1.56)	0.153	0.20 (0.04,1.14)	0.069	0.17 (0.03,0.99)	0.048*
Trend test	NA	0.59 (0.31,1.11)	0.102	0.60 (0.30,1.22)	0.161	0.49 (0.23,1.06)	0.069	0.45 (0.21,0.99)	0.047*
With mixed plaque									
TyG per SD	3 ( 5.0)	-	-	-	-	-	-	-	-
TyG_low_	2 (10)	1(reference)	1(reference)	1(reference)	1(reference)	1(reference)	1(reference)	1(reference)	1(reference)
TyG_mid_	1 (5)	0.50 (0.05,5.54)	0.574	0.54 (0.05,5.98)	0.612	1.74 (0.07,45.28)	0.738	-	0.998
TyG_high_	0 (0)	0 (0,Inf)	0.999	0 (0,Inf)	0.999	0 (0,Inf)	0.999	0 (0,Inf)	0.999
Trend test	NA	0.29 (0.04,1.96)	0.203	0.35 (0.05,2.67)	0.311	0.68 (0.07,6.31)	0.735	0.42 (0.02,7.97)	0.563
TCFA = 0									
TyG per SD	12 ( 5.9)	0.47(0.25,0.87)	0.017*	0.50(0.26,0.98)	0.043*	0.39(0.19,0.82)	0.012*	0.39(0.18,0.84)	0.017*
TyG_low_	6 (8.8)	1(reference)	1(reference)	1(reference)	1(reference)	1(reference)	1(reference)	1(reference)	1(reference)
TyG_mid_	5 (7.4)	0.81 (0.25,2.66)	0.731	0.90 (0.25,3.21)	0.875	0.93 (0.25,3.37)	0.908	0.95 (0.25,3.60)	0.934
TyG_high_	1 (1.4)	0.15 (0.02,1.29)	0.084	0.19 (0.02,1.64)	0.130	0.11 (0.01,1.12)	0.063	0.11 (0.01,1.36)	0.086
Trend test	NA	0.49 (0.23,1.07)	0.073	0.53 (0.23,1.21)	0.132	0.45 (0.19,1.07)	0.071	0.46 (0.18,1.17)	0.104
TCFA = 1									
TyG per SD	7 (10.1)	0.53(0.22,1.30)	0.164	0.54(0.17,1.72)	0.295	1.12(0.00,298.31)	0.968	Inf(0.00,inf)	0.994
TyG_low_	3 (13)	1(reference)	1(reference)	1(reference)	1(reference)	1(reference)	1(reference)	1(reference)	1(reference)
TyG_mid_	3 (13)	0.85 (0.17,4.24)	0.843	0.73 (0.1,5.14)	0.751	0.50 (0.06,4.43)	0.532	1.70 (0.10,28.36)	0.710
TyG_high_	1 (4.3)	0.26 (0.03,2.56)	0.250	0.36 (0.03,4.46)	0.424	0.25 (0.02,3.35)	0.295	0.56 (0.04,7.69)	0.662
Trend test	NA	0.57 (0.22,1.49)	0.249	0.61 (0.19,2.04)	0.426	0.50 (0.14,1.76)	0.279	0.78 (0.23,2.68)	0.698

Adjust I model adjusts for sex and age; Adjust II model adjusts for adjust I plus ejection fraction, smoke, hypertension, hyperlipidemia, diabetes mellitus and killip classification; Adjust III model adjusts for adjust II + creatine kinase, heart rate and C-reactive protein

HR, hazard ratio; CI, confidence interval; TyG, triglyceride glucose index.SD, Standard deviation; Inf, infinity; NA, Not available

*P < 0.05

The addition of the TyG index and morphological characteristics had a significant incremental effect on the AUC obtained from the four models: 0.899 vs. 0.914 vs. 0.916 vs. 0.985 (Additional file 1: Fig. S1A and Table S1). Survival ROC curves for the discriminatory value of MACEs are shown in Additional file 1: Fig. S1B (AUC = 0.751, 0.781, 0.798, and 0.846, respectively). Furthermore, we divided the group into tertiles of the prediction values among model II (P < 0.001) (Additional file 1: Fig. S1C) and model III (P < 0.001) (Additional file 1: Fig. S1D) and found a significant difference between the groups (P < 0.001). ROC analysis was performed to evaluate the diagnostic value of TyG combined with the morphological characteristics of plaques (PR&PE) in predicting MI (Additional file 1: Fig. S2). Moreover, the addition of the TyG index improved the reclassification and discrimination ability beyond the baseline risk model with a category-free net reclassification improvement (NRI) index value of 0.108 and an integrated discrimination improvement (IDI) index value of 0.108 (Additional file 1: Table S2).

## Discussion

The present study investigated the prognostic role of the TyG index combined with OCT plaque characteristics in patients with STEMI undergoing primary PCI and demonstrated that after adjustment for confounding factors, the incidence of MACEs was significantly higher among participants in the middle tertile of the TyG index in the presence of PR. However, this tendency was absent in patients with PE. In addition, the ROC curve and survival ROC curve showed that the TyG index combined with the microstructural features revealed by OCT had a higher predictive value for MACEs than traditional risk factors alone in patients with STEMI.

### TyG and coronary artery disease

Recent studies have shown that the TyG index is correlated with IR and is considered to be a surrogate marker of IR [24]. The TyG index, derived from FPG and TG levels, was found to be associated with carotid atherosclerosis and a high risk of cardiovascular disease [25, 26] and metabolic syndrome [27]. In addition, Sanchez-Inigo et al. reported that TyG might help identify individuals at high risk of a CVD event in a large and long-term follow-up cohort [28]. Several studies have demonstrated that this index is closely correlated with the progression of coronary artery calcium regardless of conventional risk factors [29] and contributes to arterial stiffness, as measured by brachial-ankle pulse wave velocity in both sexes [30]. Furthermore, in healthy participants, patients with a higher TyG index were more likely to have a greater risk of incident renal microvascular damage, including microalbuminuria and chronic kidney disease [31]. Compared with the homeostasis model assessment (HOMA) index, the TyG index was found to have a stronger association with carotid atherosclerosis [32] and carotid artery intima-media thickness [33]. The TyG index was closely related to a higher risk of cardiovascular events in patients with T2DM and ACS who underwent PCI after adjustment for other confounders [34]. Jin et al. reported that the TyG index was correlated with future cardiovascular events (including death, stroke, MI and revascularization after discharge), which indicates that the TyG index might be beneficial for predicting clinical outcomes among patients with stable coronary artery disease [35]. In the cohort of non-ST-segment elevation ACS, Mao et al. [12] showed that the TyG index was a helpful and independent predictor of the composite of the endpoints.

### Prognostic value of TyG combined with plaque morphology characteristics

While the relationship between glucose metabolism and PE remains unclear, patients with diabetes mellitus were found to have a higher prevalence of PR on pathology [36], more frequent macrophage accumulation, higher lipid burden, greater maximal lipid arc, thinner fibrous cap thickness, and higher prevalence of thin-cap fibroatheroma at the culprit plaques, indicating a higher level of pan-vascular instability [37]. Previous studies comparing the influence of PE and PR in acute MI demonstrated that despite similar clinical characteristics, patients with PE and admission hyperglycaemia and insulin resistance showed larger infarct sizes [38], more multi-vessel lesions [39], and a higher occurrence of the no-reflow phenomenon than normoglycaemic patients. In the present study, we compared the predictive ability of the TyG index combined with plaque morphology (PR or PE). The difference in the prognostic value of the TyG index between the PR and PE groups was confirmed after adjustment for traditional risk factors. The results indicated that the HR for MACEs was significantly higher in patients in the middle tertile of TyG than in those in the low tertile of TyG after full additional adjustment (HR, 5.45; 95% CI, 1.10–27.09; P = 0.038). However, being in the high tertile of TyG independently and significantly increased the risk of major bleeding events (HR, 2.50; 95% CI, 1.11–5.65; P = 0.028) among patients with PE. This underlines the importance of lipid and glucose metabolism, as revealed by the TyG index, in the mechanism of PR and PE development. Our results provide strong clinical evidence for the greater predictive value of MACEs in relation to the TyG index in patients with PR than in those with PE. A recent study on patients with STEMI has shown that random plasma glucose on admission and glucose variable tendency are risk factors for PR but not PE [40]. Therefore, we presume that targeting insulin resistance may be beneficial for patients with STEMI and PR by reducing the thrombus and lipid burdens.

### Mechanism

Insulin resistance at admission in participants with acute MI plays a key role in enhancing local platelet activation and thrombin generation [41] and is associated with a large thrombus burden, resulting in adverse cardiac outcomes [42]. Insulin resistance and glycaemic disorders, characterized by hyperglycaemia with hyperinsulinaemia or normoglycaemia, induce various pathophysiological abnormalities and are therefore accompanied by several cardiovascular and metabolic risk factors and comorbidities. Previous studies have found that patients with higher insulin resistance might have more pronounced concentric cardiac remodelling and a higher incidence of arterial stiffness [43]. However, the potential pathologic mechanisms that determine these correlations have still not been completely clarified [44]. Previous studies have reported that hyperinsulinaemia is usually accompanied by increased oxidative stress, systemic and tissue inflammation, higher free fatty acid levels, imbalances in glucose and lipid metabolism, and activation of the renin–angiotensin–aldosterone system and sympathetic nervous system [45, 46]. The effects are initiated by increased intracellular calcium, ultimately resulting in cell damage, increased collagen and glaciation end products, and cellular hypertrophy and fibrosis [47]. Interestingly, a prior study showed that insulin resistance plays a key role in regulating matrix metalloproteinases, HDL-C levels, and TG levels, which trigger functional and structural modifications of the cardiovascular system [48]. Notably, the above-mentioned process affects the entire cardiovascular system, not only the heart. Moreover, by inducing a pro-inflammatory and oxidative state [49] and increasing levels of sFas apoptosis [50] and CD14 (bright)/CD16 ( +) monocyte chemoattractant protein [51], the statuses of insulin resistance, dynamic glucose fluctuation, and hyperglycaemia are closely correlated with plaque rupture and result in a poor prognosis in both non-diabetic and diabetic patients with acute MI [52–55].

### Limitations

First, this was a single-centre study that was restricted to a selected group of Chinese participants, and the time of follow-up might not be long enough. Therefore, further prospective, multi-centre studies are needed to verify the findings of the present study. Second, the TyG index was assessed only once at admission, and its fluctuations were not measured or analysed during the follow-up period. Third, the results are likely to be influenced by confounders associated with the presence of the disease due to the selection of participants on the basis of disease findings. Although we controlled for confounders in the adjustment of the model to reduce bias, a residual selection bias remained. Furthermore, this was a single-centre study with a relatively small sample size, and patients with congestive heart failure, extremely tortuous or heavily calcified vessels, cardiac shock, chronic total occlusion, serious liver dysfunction, allergy to contrast media, and severe hepatic and renal insufficiency were excluded; thus, selection bias may exist. An independent study with a larger sample size is warranted to verify our study results. Moreover, diabetes mellitus and hyperlipidaemia therapy, which might have had a potential impact on the TyG index and study results, was not assessed. Finally, compared with the OCTAMI registry study, which focuses on cardiovascular studies, other glucose-related variables (such as the type of diabetic drugs, HOMA index, and insulin resistance index level) were not assessed in this study.

## Conclusion

The results of this study suggest that OCT microstructural features of culprit lesions in combination with the TyG index, a surrogate estimate of insulin resistance, can be used in clinical practice to support risk stratification and predict a poorer prognosis in patients with STEMI.

## Sponsor’s role

This study was supported by the Chinese Academy of Medical Sciences Innovation Fund for Medical Sciences (2016-I2M-1-009), National Natural Science Funds (number: 81970308) and the Fund of "Sanming" Project of Medicine in Shenzhen (number: SZSM201911017).

## Supplementary Information

**Additional file 1: Figure S1.** ROC curve. Model 1, traditional risk factors including sex, age, ejection fraction, hypertension, hyperlipidemia, diabetes mellitus, history of PCI, creatine kinase, C-reactive protein, low density lipoprotein, Model 2, model 1 plus TyG; Model 3, model 2 plus plaque rupture and plaque erosion; Model 2, model 1 plus microstructural features of culprit lesion by OCT including TCFA, FCT, max lipid-arc, minimal lumen area, macrophage, thrombus, healing plaque, micro-vessels, cholesterol crystal, calcification, micro- calcification, mixed plaque, lipid plaque, fibrous plaque; SFig. 4B, Survival ROC curve, the confounding factors of model I, II, III,IV are as same as the Sfig. 4A; SFig 4C, Kaplan–Meier curves showing cumulative MACE rates stratified by the tertiles level of TyG among Model II; SFig 4D, Kaplan–Meier curves showing cumulative MACE rates stratified by the tertiles level of TyG among Model III. *AUC, areas under the ROC curve; CI, 95% confidence interval.*.** Figure S2**. Receiver operating characteristic curve of triglyceride glucose index combined with plaque characteristics for predicting MI. The area under the ROC was 0.855 (95% CI, 0.807–0.894). The Youden index was 0.694 and corresponding sensitivity and specificity were 75.00% and 94.36%. *MI, myocardial infarction; AUC, areas under the ROC curve; CI, 95% confidence interval.*

## Data Availability

The datasets used and/or analyzed during this study are available from the corresponding author on reasonable request.

## Abbreviations

TyG: Triglyceride glucose
STEMI: ST-elevated myocardial infarction
OCT: Optical coherence tomography
MACEs: Major adverse cardiovascular events
MI: Myocardial infarction
PR: Plaque rupture
PE: Plaque erosion
HR: Hazard ratio
CI: Confidence interval
ROC: Receiver operating characteristic
IR: Insulin resistance
TG: Triglycerides
FPG: Fasting plasma glucose
PCI: Primary percutaneous coronary intervention
OCTAMI: Optical Coherence Tomography Examination in Acute Myocardial Infarction
eGFR: Estimated glomerular filtration rate
IWG-IVOCT: International Working Group for Intravascular Optical Coherence Tomography
TC: Total cholesterol
LPA: Lipse activator
HDL-C: High-density lipoprotein cholesterol
NRI: Net reclassification improvement
IDI: Integrated discrimination improvement
AUC: Area under the curve
