# Supplementary material for: Triglyceride glucose index combined with plaque characteristics as a novel biomarker for cardiovascular outcomes after percutaneous coronary intervention in ST-elevated myocardial infarction patients: an intravascular optical coherence tomography study
Source: Cardiovasc Diabetol. 2021 Jun 28;20:131. doi: 10.1186/s12933-021-01321-7 (PMC8240222; doi:10.1186/s12933-021-01321-7)
Supplement: Supplementary file 1 — Additional file 1: Figure S1. ROC curve. Model 1, traditional risk factors including sex, age, ejection fraction, hypertension, hyperlipidemia, diabetes mellitus, history of PCI, creatine kinase, C-reactive protein, low density lipoprotein, Model 2, model 1 plus TyG; Model 3, model 2 plus plaque rupture and plaque erosion; Model 2, model 1 plus microstructural features of culprit lesion by OCT including TCFA, FCT, max lipid-arc, minimal lumen area, macrophage, thrombus, healing plaque, micro-vessels, cholesterol crystal, calcification, micro- calcification, mixed plaque, lipid plaque, fibrous plaque; SFig. 4B, Survival ROC curve, the confounding factors of model I, II, III,IV are as same as the Sfig. 4A; SFig 4C, Kaplan–Meier curves showing cumulative MACE rates stratified by the tertiles level of TyG among Model II; SFig 4D, Kaplan–Meier curves showing cumulative MACE rates stratified by the tertiles level of TyG among Model III. AUC, areas under the ROC curve; CI, 95% confidence interval.. Figure S2. Receiver operating characteristic curve of triglyceride glucose index combined with plaque characteristics for predicting MI. The area under the ROC was 0.855 (95% CI, 0.807–0.894). The Youden index was 0.694 and corresponding sensitivity and specificity were 75.00% and 94.36%. MI, myocardial infarction; AUC, areas under the ROC curve; CI, 95% confidence interval. [file 12933_2021_1321_MOESM1_ESM.docx]

Additional file 1: Table S1 C-statistics for discrimination ability of various models

| Model variables | AUC | 95% CI | P value | Youden index | Sensitivity | Specificity | Z value | P for comparison |
| --- | --- | --- | --- | --- | --- | --- | --- | --- |
| Model 1: Baseline risk model^a^ | 0.899 | 0.858, 0.932 | <0.0001 | 0.6908 | 89.47 | 79.61 | Reference | Reference |
| Model 2: Model 1+ TyG index^b^ | 0.914 | 0.875, 0.945 | <0.0001 | 0.7474 | 94.74 | 80.00 | 0.897 | 0.3696 |
| Model 3: Model 2+ plaque rupture/erosion^c^ | 0.916 | 0.877, 0.946 | <0.0001 | 0.7434 | 94.74 | 79.61 | 0.995 | 0.3196 |
| Model 4: Model 3+ microstructural features of plaques^d^ | 0.985 | 0.962, 0.996 | <0.0001 | 0.8751 | 89.47 | 98.04 | 3.123 | 0.0018* |

Italic values indicate statistically signifcant associations

*TyG triglyceride glucose, AUC area under the curve, CI confdence interval*

^a^ The baseline risk model includes gender, age, ejection fraction, hypertension, hyperlipidemia, diabetes mellitus, history of PCI, creatine kinase, C-reactive protein, low density lipoprotein.

^b^ Model 2, model 1 plus TyG

^c^ Model 3, model 2 plus plaque rupture and plaque erosion

^d^ Model 2, model 1 plus microstructural features of culprit lesion by OCT including TCFA, FCT, max lipid-arc, minimal lumen area, macrophage, thrombus, healing plaque, micro-vessels, cholesterol crystal, calcification, micro- calcification, mixed plaque, lipid plaque, fibrous plaque

*P<0.05.

Additional file 1: Table S2 Category-free NRI and IDI for the incremental predictive values of various models

| Model variables | Category-free NRI | | | IDI | | |
| --- | --- | --- | --- | --- | --- | --- |
|  | NRI Index | 95% CI | P value | Index | 95% CI | P value |
| Model 1: Baseline risk model^a^ | - | - | Reference | - | - | Reference |
| Model 2: Model 1+ TyG index^b^ | 0.108 | -0.128,0.344 | 0.369 | 0.108 | -0.134,0.350 | 0.382 |
| Model 3: Model 2+ plaque rupture/erosion^c^ | 0.120 | -0.116,0.177 | 0.320 | 0.120 | -0.122,0.362 | 0.319 |
| Model 4: Model 3+ microstructural features of plaques^d^ | 0.183 | -0.028,0.395 | 0.089 | 0.184 | -0.033,0.400 | 0.097 |

*TyG triglyceride glucose, NRI net reclassification improvement, IDI integrated discrimination improvement, CI confidence interval*

^a^ The baseline risk model includes gender, age, ejection fraction, hypertension, hyperlipidemia, diabetes mellitus, history of PCI, creatine kinase, C-reactive protein, low density lipoprotein

^b^ Model 2, model 1 plus TyG

^c^ Model 3, model 2 plus plaque rupture and plaque erosion

^d^ Model 2, model 1 plus microstructural features of culprit lesion by OCT including TCFA, FCT, max lipid-arc, minimal lumen area, macrophage, thrombus, healing plaque, micro-vessels, cholesterol crystal, calcification, micro- calcification, mixed plaque, lipid plaque, fibrous plaque

*P<0.05.

| 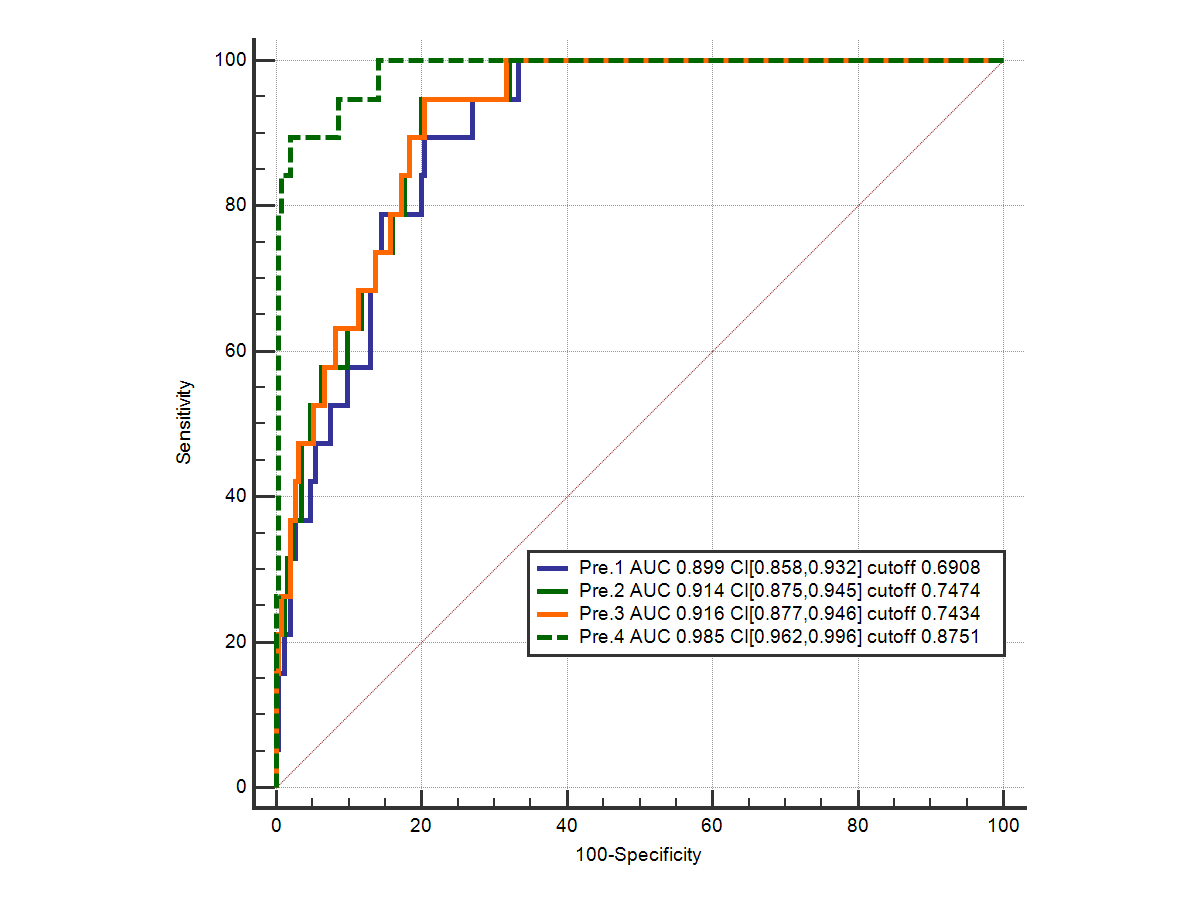 | 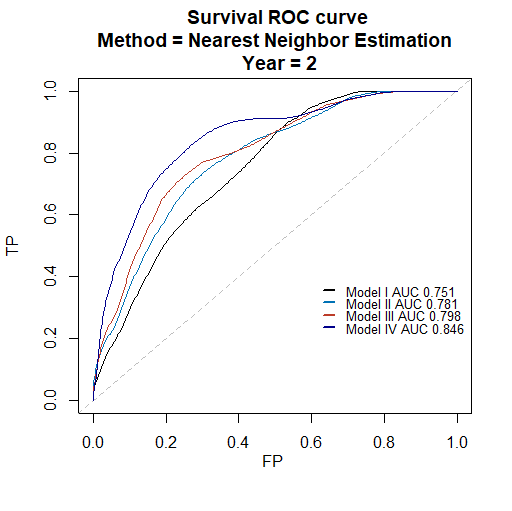 |
| --- | --- |
| Additional file 1: Figure S1A ROC curve | Additional file 1: Figure S1B Survival ROC curve |
| 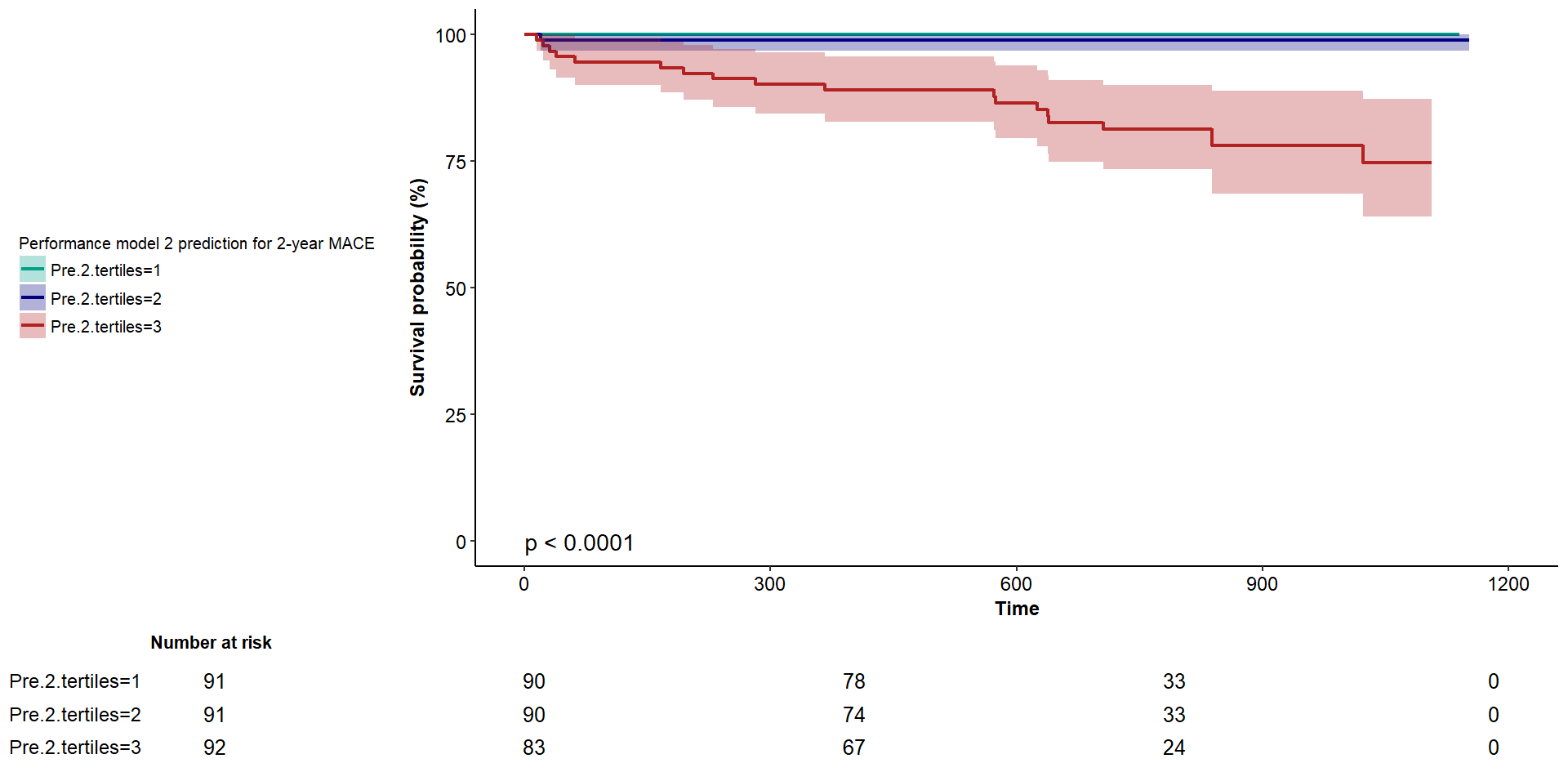 | 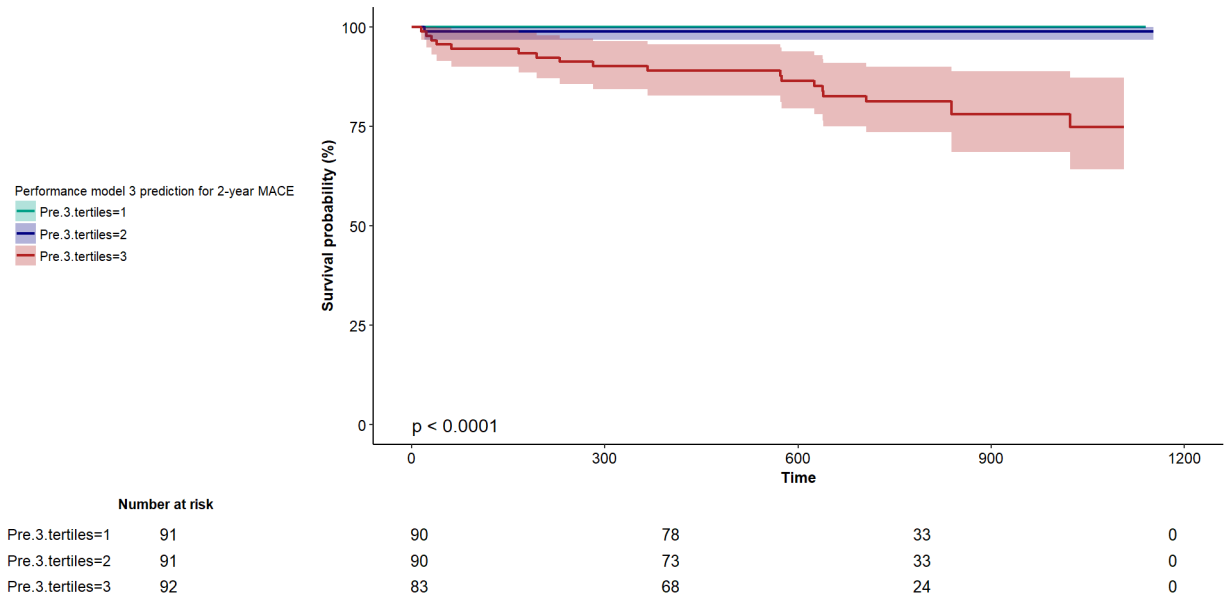 |
| Additional file 1: Figure S1C Performance model II prediction tertiles for 2-year MACE | Additional file 1: Figure S1D Performance model III prediction tertiles for 2-year MACE |

Additional file 1: Figure S1A, ROC curve

Model 1, traditional risk factors including sex, age, ejection fraction, hypertension, hyperlipidemia, diabetes mellitus, history of PCI, creatine kinase, C-reactive protein, low density lipoprotein,

Model 2, model 1 plus TyG

Model 3, model 2 plus plaque rupture and plaque erosion

Model 4, model 1 plus microstructural features of culprit lesion by OCT including TCFA, FCT, max lipid-arc, minimal lumen area, macrophage, thrombus, healing plaque, micro-vessels, cholesterol crystal, calcification, micro- calcification, mixed plaque, lipid plaque, fibrous plaque

Figure 1B, Survival ROC curve, the confounding factors of model I, II, III ,IV are as same as the fig. 3A.

Figure 1C, Kaplan-Meier curves showing cumulative MACE rates stratified by the tertiles level of TyG among Model II.

Figure 1D, Kaplan-Meier curves showing cumulative MACE rates stratified by the tertiles level of TyG among Model III

*AUC, areas under the ROC curve; CI, 95% confidence interval*


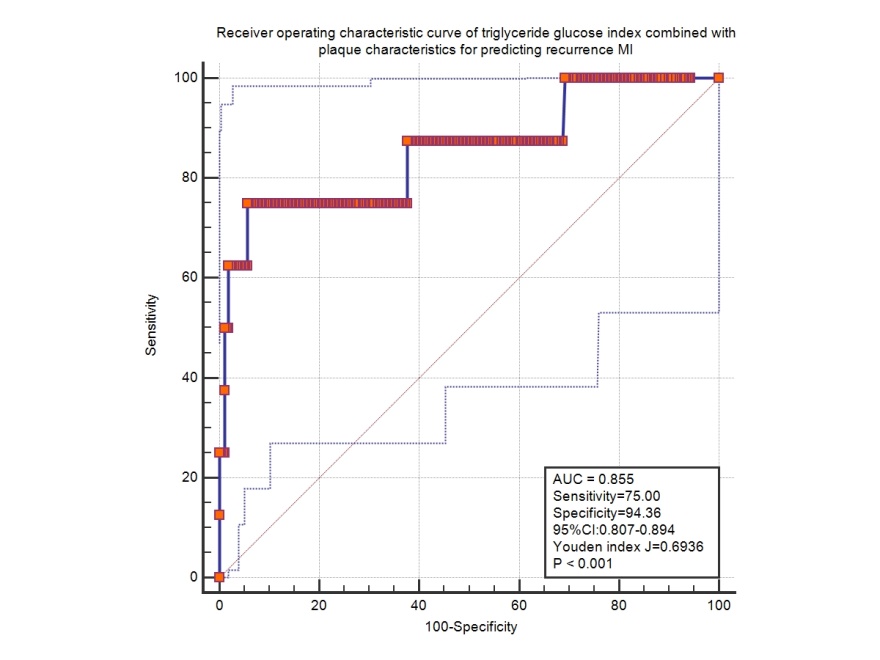


Additional file 1: Figure S2, Receiver operating characteristic curve of triglyceride glucose index combined with plaque characteristics for predicting MI.

*MI, myocardial infarction; AUC, areas under the ROC curve; CI, 95% confidence interval*
